# 2,4-Dibromo-2,3-dihydro-1*H*-inden-1-yl acetate

**DOI:** 10.1107/S1600536812023173

**Published:** 2012-05-26

**Authors:** Ísmail Çelik, Mehmet Akkurt, Makbule Yilmaz, Ahmet Tutar, Ramazan Erenler, Canan Kazak

**Affiliations:** a Department of Physics, Faculty of Arts and Sciences, Cumhuriyet University, 58140 Sivas, Turkey; b Department of Physics, Faculty of Sciences, Erciyes University, 38039 Kayseri, Turkey; c Department of Chemistry, Faculty of Art and Science, Sakarya University, 54187 Adapazarı, Turkey; d Department of Chemistry, Faculty of Art and Science, Gaziosmanpaşa University, 60240 Tokat, Turkey; e Department of Physics, Faculty of Arts and Sciences, Ondokuz Mayıs University, 55139 Samsun, Turkey

## Abstract

In the title compound, C_11_H_10_Br_2_O_2_, the cyclo­pentene ring fused to the benzene ring adopts an envelope conformation, with the C atom attached to the Br atom as the flap. The crystal structure does not exhibit any classical hydrogen bonds. The mol­ecular packing is stabilized by van der Waals forces and π–π stacking inter­actions with a centroid–centroid distance of 3.811 (4) Å.

## Related literature

For bromination of hydro­carbons, see: Catto *et al.* (2010 ▶); Erenler & Çakmak (2004 ▶); Erenler *et al.* (2006 ▶); McClure *et al.* (2011 ▶); Mitrochkine *et al.* (1995 ▶); Snyder & Brill (2011 ▶); Wu (2006 ▶); Çakmak *et al.* (2006 ▶). For puckering parameters, see: Cremer & Pople (1975 ▶).
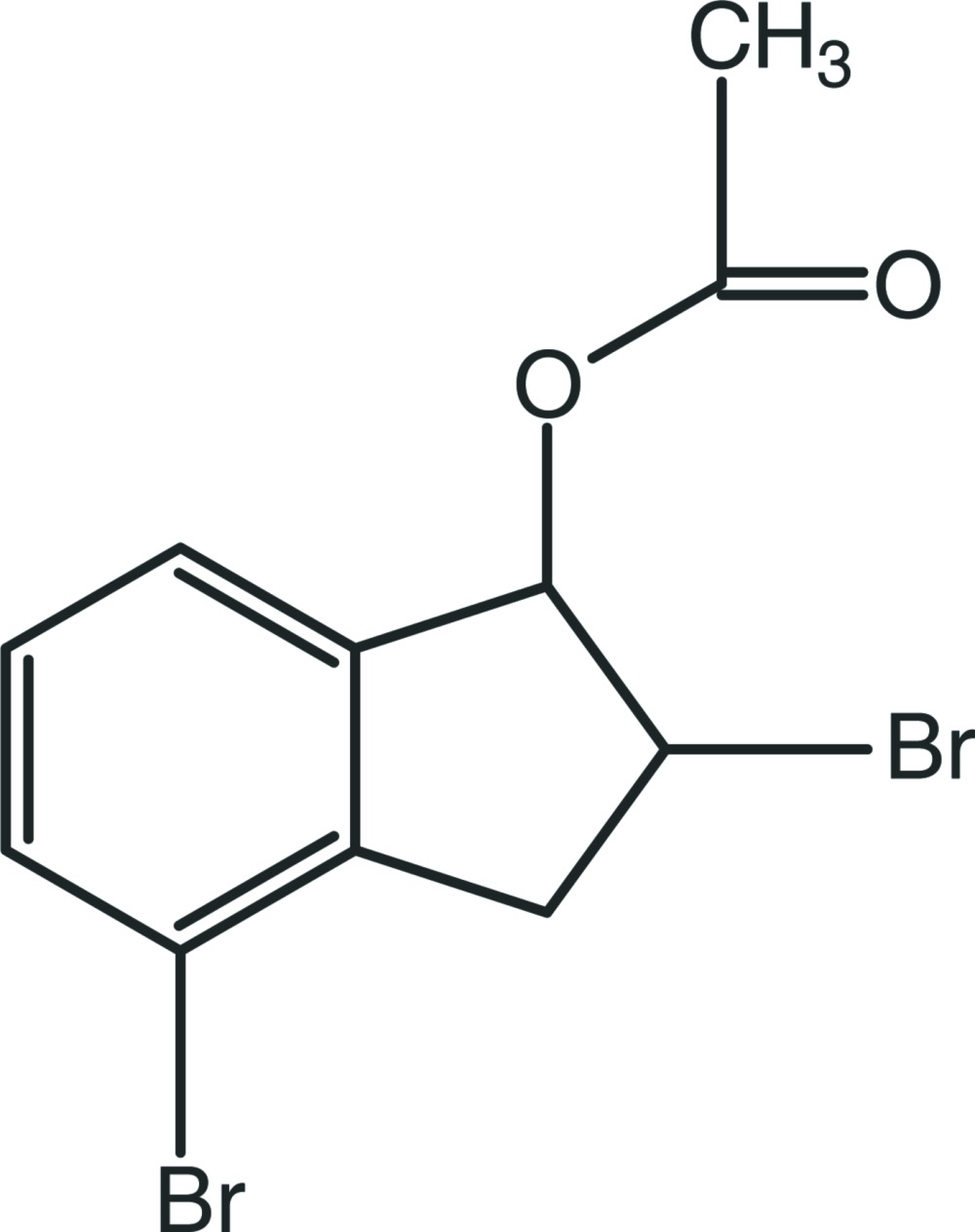


## Experimental

### 

#### Crystal data

C_11_H_10_Br_2_O_2_
*M* *_r_* = 333.99
Triclinic, 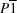

*a* = 8.1423 (7) Å
*b* = 8.6891 (9) Å
*c* = 9.0028 (8) Å
α = 76.163 (8)°
β = 68.105 (7)°
γ = 86.397 (8)°
*V* = 573.60 (10) Å^3^
*Z* = 2
Mo *K*α radiation
μ = 7.04 mm^−1^
*T* = 296 K
0.43 × 0.35 × 0.28 mm

#### Data collection

Stoe IPDS 2 diffractometer
Absorption correction: integration (*X-RED32*; Stoe & Cie, 2002 ▶) *T*_min_ = 0.152, *T*_max_ = 0.243
6542 measured reflections
2635 independent reflections
1958 reflections with *I* > 2σ(*I*)
*R*_int_ = 0.110

#### Refinement

*R*[*F* ^2^ > 2σ(*F* ^2^)] = 0.067
*wR*(*F* ^2^) = 0.178
*S* = 1.02
2635 reflections
137 parameters
H-atom parameters constrained
Δρ_max_ = 1.20 e Å^−3^
Δρ_min_ = −1.42 e Å^−3^

### 

Data collection: *X-AREA* (Stoe & Cie, 2002 ▶); cell refinement: *X-AREA*; data reduction: *X-RED32* (Stoe & Cie, 2002 ▶); program(s) used to solve structure: *SIR97* (Altomare *et al.*, 1999 ▶); program(s) used to refine structure: *SHELXL97* (Sheldrick, 2008 ▶); molecular graphics: *ORTEP-3 for Windows* (Farrugia, 1997 ▶); software used to prepare material for publication: *WinGX* (Farrugia, 1999 ▶) and *PLATON* (Spek, 2009 ▶).

## Supplementary Material

Crystal structure: contains datablock(s) global, I. DOI: 10.1107/S1600536812023173/fj2559sup1.cif

Structure factors: contains datablock(s) I. DOI: 10.1107/S1600536812023173/fj2559Isup2.hkl

Supplementary material file. DOI: 10.1107/S1600536812023173/fj2559Isup3.cml

Additional supplementary materials:  crystallographic information; 3D view; checkCIF report

## Figures and Tables

**Fig. 1.**
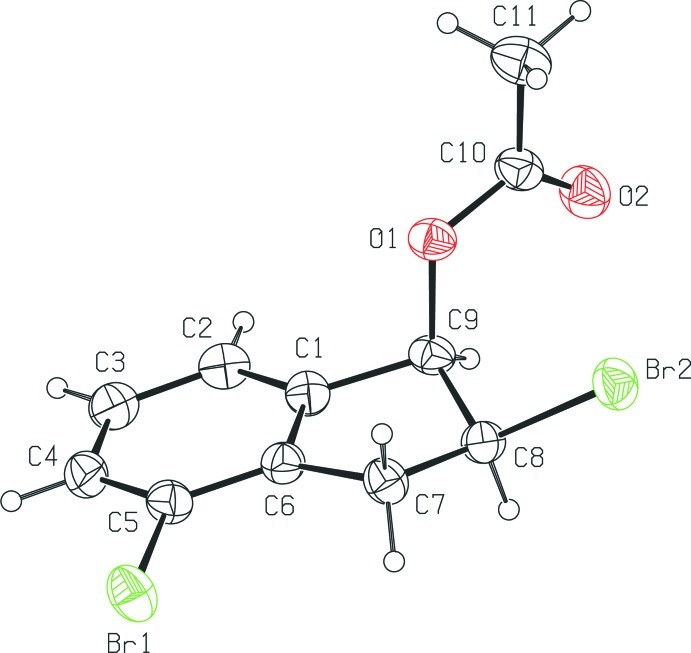
An ORTEP drawing of the title compound with the atom numbering scheme. Displacement ellipsoids for non-H atoms are drawn at the 30% probability level.

**Fig. 2.**
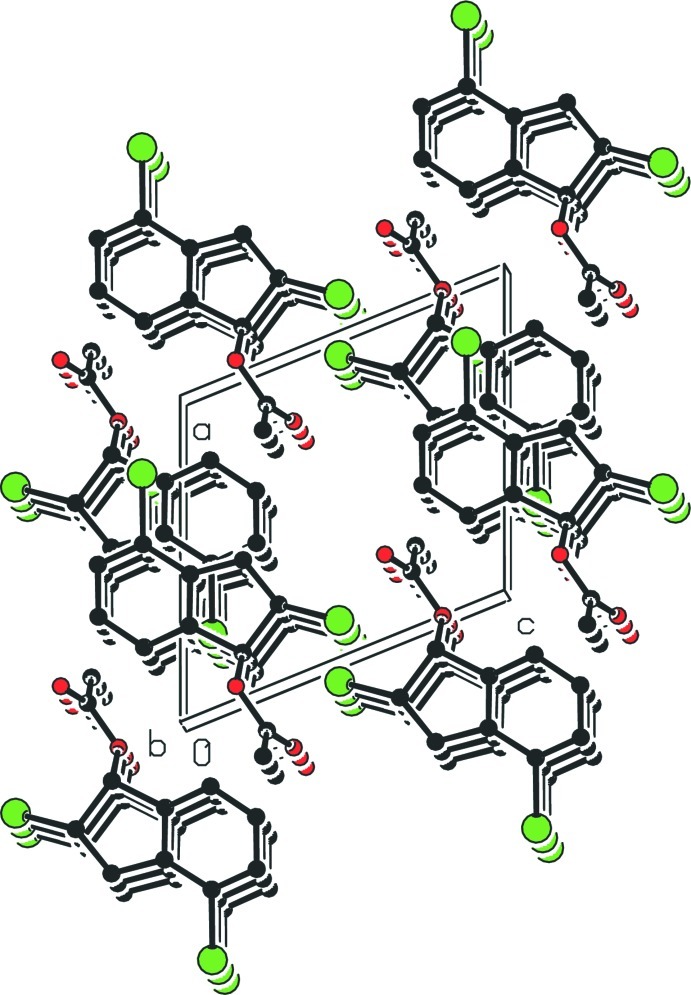
View of the packing of the title compound, along the b axis. H atoms are omitted for the sake of clarity.

## supplementary crystallographic information

### Comment

Brominations of hydrocarbons are important processes in synthetic chemistry (Çakmak *et al.*, 2006; Erenler *et al.*, 2006; Erenler & Çakmak, 2004). Indanes are important class of molecules due to the pharmacological and medicinal properties (Mitrochkine *et al.*, 1995; Catto *et al.*, 2010; Wu, 2006; McClure *et al.*, 2011) as well as natural product chemistry (Snyder & Brill, 2011).

The five-membered C1C6–C9 cyclopentene ring in the title compound, (Fig. 1), exhibits an envelope-shaped conformation, with the C8 atom attached to Br2 atom at the flap [the puckering parameters (Cremer & Pople, 1975) Q(2) = 0.279 (7) Å, φ(2) = 290.5 (13) °]. The Br1–C5–C6–C1, Br2–C8–C9–C1, C9–O1–C10–C11 and C9–O1–C10–O2 torsion angles are -178.0 (4), -152.1 (4), -170.2 (6) and 10.3 (9) °, respectively.

In the crystal, there is no classic hydrogen bonds. The crystal structure is stabilized by van der Waals forces and π-π stacking interactions [*Cg*2···*Cg*2(1 - *x*, 1 - *y*, 2 - *z*) = 3.811 (4) Å] between the centroids (*Cg*2) of the benzene rings of the adjacent molecules. Fig. 2 shows the molecular packing of the title compound along the *b* axis.

### Experimental

To a cooled solution (273 K) of 2,4-dibromo-1-hyodroxyindane (0.2 g, 0.68 mmol) in pyridine (6.0 ml) was added acetic anhydride (1.0 ml) dropwise. After completion of the reaction for 4 h at room temperature, the solvent was removed under reduced pressure to form the solid product which was crystalized from dichloromethane/hexane to yield the 1-acetate-2,4-dibromo-indane (0.21 g, 95%). ^1^H-NMR (300 MHz, CDCl_3_) δ 7.40–7.60 (m, 3H), 6.0 (d, 1H), 4.90 (dt, 1H), 3.50 (m, 2H), 2.20 (s, 3H).

### Refinement

The hydrogen atoms were placed in calculated positions (C—H = 0.93–0.98 Å) and refined as riding atoms with *U*_iso_(H) = 1.2*U*_eq_(C) or 1.5*U*_eq_(methyl C). Eight poorly fitted reflections (0 1 0), (-2 0 2), (2 - 1 4), (-5 - 8 2), (3 - 1 3), (1 - 1 4), (0 2 1) and (-1 0 2) were omitted from the refinement. The highest residual peak and the deepest hole are located 0.93 and 0.89 Å, respectively, from atom Br2.

### Crystal data

**Table:**

C_11_H_10_Br_2_O_2_	*Z* = 2
*M**_r_* = 333.99	*F*(000) = 324
Triclinic, *P*1	*D*_x_ = 1.934 Mg m^−^^3^
Hall symbol: -P 1	Mo *K*α radiation, λ = 0.71073 Å
*a* = 8.1423 (7) Å	Cell parameters from 9714 reflections
*b* = 8.6891 (9) Å	θ = 2.4–28.1°
*c* = 9.0028 (8) Å	µ = 7.04 mm^−^^1^
α = 76.163 (8)°	*T* = 296 K
β = 68.105 (7)°	Prism, colourless
γ = 86.397 (8)°	0.43 × 0.35 × 0.28 mm
*V* = 573.60 (10) Å^3^	

### Data collection

**Table:**

Stoe IPDS 2 diffractometer	2635 independent reflections
Radiation source: sealed X-ray tube, 12 x 0.4 mm long-fine focus	1958 reflections with *I* > 2σ(*I*)
Plane graphite monochromator	*R*_int_ = 0.110
Detector resolution: 6.67 pixels mm^-1^	θ_max_ = 27.5°, θ_min_ = 2.5°
ω scans	*h* = −10→10
Absorption correction: integration (*X-RED32*; Stoe & Cie, 2002)	*k* = −11→10
*T*_min_ = 0.152, *T*_max_ = 0.243	*l* = −11→11
6542 measured reflections	

### Refinement

**Table:**

Refinement on *F*^2^	Primary atom site location: structure-invariant direct methods
Least-squares matrix: full	Secondary atom site location: difference Fourier map
*R*[*F*^2^ > 2σ(*F*^2^)] = 0.067	Hydrogen site location: inferred from neighbouring sites
*wR*(*F*^2^) = 0.178	H-atom parameters constrained
*S* = 1.02	*w* = 1/[σ^2^(*F*_o_^2^) + (0.1053*P*)^2^] where *P* = (*F*_o_^2^ + 2*F*_c_^2^)/3
2635 reflections	(Δ/σ)_max_ < 0.001
137 parameters	Δρ_max_ = 1.20 e Å^−^^3^
0 restraints	Δρ_min_ = −1.42 e Å^−^^3^

### Special details

**Table:**

Geometry. Bond distances, angles *etc*. have been calculated using the rounded fractional coordinates. All su's are estimated from the variances of the (full) variance-covariance matrix. The cell e.s.d.'s are taken into account in the estimation of distances, angles and torsion angles
Refinement. Refinement on *F*^2^ for ALL reflections except those flagged by the user for potential systematic errors. Weighted *R*-factors *wR* and all goodnesses of fit *S* are based on *F*^2^, conventional *R*-factors *R* are based on *F*, with *F* set to zero for negative *F*^2^. The observed criterion of *F*^2^ > σ(*F*^2^) is used only for calculating -*R*-factor-obs *etc*. and is not relevant to the choice of reflections for refinement. *R*-factors based on *F*^2^ are statistically about twice as large as those based on *F*, and *R*-factors based on ALL data will be even larger.

### Fractional atomic coordinates and isotropic or equivalent isotropic displacement parameters (Å^2^)

**Table:**

	*x*	*y*	*z*	*U*_iso_*/*U*_eq_	
Br1	0.22277 (8)	0.18390 (9)	1.10206 (9)	0.0641 (3)	
Br2	0.91526 (9)	0.24936 (9)	0.49626 (8)	0.0579 (3)	
O1	0.9902 (5)	0.1989 (5)	0.8133 (5)	0.0464 (11)	
O2	1.2454 (6)	0.3332 (6)	0.6377 (6)	0.0625 (16)	
C1	0.7425 (7)	0.3340 (6)	0.9593 (7)	0.0433 (17)	
C2	0.7505 (8)	0.3703 (7)	1.0998 (8)	0.0495 (17)	
C3	0.6006 (10)	0.3485 (8)	1.2402 (8)	0.0549 (19)	
C4	0.4421 (9)	0.2927 (8)	1.2408 (8)	0.0527 (19)	
C5	0.4366 (8)	0.2609 (7)	1.1011 (8)	0.0497 (17)	
C6	0.5867 (7)	0.2826 (7)	0.9560 (7)	0.0438 (17)	
C7	0.6106 (7)	0.2538 (7)	0.7923 (7)	0.0472 (17)	
C8	0.7877 (7)	0.3406 (7)	0.6838 (7)	0.0446 (17)	
C9	0.8911 (7)	0.3415 (6)	0.7950 (7)	0.0425 (14)	
C10	1.1652 (8)	0.2097 (8)	0.7179 (8)	0.0488 (17)	
C11	1.2441 (10)	0.0501 (9)	0.7271 (11)	0.065 (3)	
H2	0.85540	0.40870	1.09820	0.0590*	
H3	0.60410	0.37060	1.33510	0.0660*	
H4	0.34100	0.27740	1.33620	0.0630*	
H7A	0.51600	0.29880	0.75610	0.0570*	
H7B	0.61690	0.14150	0.79440	0.0570*	
H8	0.76310	0.45080	0.64130	0.0530*	
H9	0.96600	0.43760	0.75890	0.0510*	
H11A	1.18960	−0.01350	0.68340	0.0970*	
H11B	1.22470	−0.00010	0.83970	0.0970*	
H11C	1.36900	0.06100	0.66390	0.0970*	

### Atomic displacement parameters (Å^2^)

**Table:**

	*U*^11^	*U*^22^	*U*^33^	*U*^12^	*U*^13^	*U*^23^
Br1	0.0461 (4)	0.0769 (5)	0.0653 (5)	−0.0077 (3)	−0.0103 (3)	−0.0237 (4)
Br2	0.0559 (4)	0.0717 (5)	0.0471 (4)	0.0049 (3)	−0.0153 (3)	−0.0228 (3)
O1	0.045 (2)	0.0381 (19)	0.056 (2)	0.0031 (15)	−0.0190 (17)	−0.0109 (17)
O2	0.051 (2)	0.061 (3)	0.067 (3)	−0.006 (2)	−0.014 (2)	−0.010 (2)
C1	0.048 (3)	0.036 (3)	0.046 (3)	0.001 (2)	−0.016 (2)	−0.012 (2)
C2	0.054 (3)	0.042 (3)	0.059 (3)	0.001 (2)	−0.026 (3)	−0.015 (3)
C3	0.070 (4)	0.051 (3)	0.053 (3)	0.013 (3)	−0.029 (3)	−0.022 (3)
C4	0.061 (4)	0.051 (3)	0.043 (3)	0.012 (3)	−0.015 (3)	−0.015 (3)
C5	0.051 (3)	0.043 (3)	0.052 (3)	0.008 (2)	−0.016 (2)	−0.012 (2)
C6	0.046 (3)	0.043 (3)	0.043 (3)	0.006 (2)	−0.018 (2)	−0.010 (2)
C7	0.040 (3)	0.052 (3)	0.049 (3)	−0.002 (2)	−0.014 (2)	−0.014 (3)
C8	0.046 (3)	0.045 (3)	0.045 (3)	0.005 (2)	−0.019 (2)	−0.012 (2)
C9	0.040 (2)	0.033 (2)	0.053 (3)	0.0027 (19)	−0.017 (2)	−0.008 (2)
C10	0.042 (3)	0.056 (3)	0.055 (3)	0.007 (2)	−0.022 (2)	−0.020 (3)
C11	0.062 (4)	0.058 (4)	0.086 (5)	0.012 (3)	−0.031 (4)	−0.034 (4)

### Geometric parameters (Å, º)

**Table:**

Br1—C5	1.900 (7)	C7—C8	1.528 (9)
Br2—C8	1.946 (6)	C8—C9	1.531 (8)
O1—C9	1.446 (7)	C10—C11	1.489 (11)
O1—C10	1.357 (8)	C2—H2	0.9300
O2—C10	1.210 (9)	C3—H3	0.9300
C1—C2	1.400 (9)	C4—H4	0.9300
C1—C6	1.384 (9)	C7—H7A	0.9700
C1—C9	1.512 (8)	C7—H7B	0.9700
C2—C3	1.374 (10)	C8—H8	0.9800
C3—C4	1.405 (11)	C9—H9	0.9800
C4—C5	1.368 (9)	C11—H11A	0.9600
C5—C6	1.399 (9)	C11—H11B	0.9600
C6—C7	1.493 (8)	C11—H11C	0.9600
			
C9—O1—C10	116.4 (5)	C1—C2—H2	121.00
C2—C1—C6	121.9 (6)	C3—C2—H2	121.00
C2—C1—C9	128.1 (6)	C2—C3—H3	120.00
C6—C1—C9	110.0 (5)	C4—C3—H3	120.00
C1—C2—C3	118.9 (6)	C3—C4—H4	120.00
C2—C3—C4	120.1 (6)	C5—C4—H4	120.00
C3—C4—C5	120.0 (6)	C6—C7—H7A	112.00
Br1—C5—C4	120.1 (5)	C6—C7—H7B	111.00
Br1—C5—C6	118.7 (5)	C8—C7—H7A	112.00
C4—C5—C6	121.3 (6)	C8—C7—H7B	112.00
C1—C6—C5	117.8 (6)	H7A—C7—H7B	109.00
C1—C6—C7	112.1 (5)	Br2—C8—H8	108.00
C5—C6—C7	130.1 (6)	C7—C8—H8	108.00
C6—C7—C8	101.3 (5)	C9—C8—H8	108.00
Br2—C8—C7	112.1 (4)	O1—C9—H9	112.00
Br2—C8—C9	113.7 (4)	C1—C9—H9	112.00
C7—C8—C9	107.2 (5)	C8—C9—H9	112.00
O1—C9—C1	106.8 (4)	C10—C11—H11A	109.00
O1—C9—C8	111.9 (5)	C10—C11—H11B	109.00
C1—C9—C8	101.4 (5)	C10—C11—H11C	109.00
O1—C10—O2	124.1 (6)	H11A—C11—H11B	109.00
O1—C10—C11	110.8 (6)	H11A—C11—H11C	109.00
O2—C10—C11	125.0 (7)	H11B—C11—H11C	109.00
			
C9—O1—C10—C11	−170.2 (6)	C2—C3—C4—C5	−0.4 (11)
C10—O1—C9—C1	−150.4 (5)	C3—C4—C5—C6	0.1 (10)
C10—O1—C9—C8	99.5 (6)	C3—C4—C5—Br1	179.5 (5)
C9—O1—C10—O2	10.3 (9)	Br1—C5—C6—C7	−0.3 (9)
C9—C1—C2—C3	−177.0 (6)	C4—C5—C6—C1	1.4 (9)
C2—C1—C6—C5	−2.7 (9)	C4—C5—C6—C7	179.2 (6)
C2—C1—C6—C7	179.2 (5)	Br1—C5—C6—C1	−178.0 (4)
C6—C1—C2—C3	2.5 (9)	C1—C6—C7—C8	−15.9 (7)
C6—C1—C9—O1	−99.5 (6)	C5—C6—C7—C8	166.3 (6)
C6—C1—C9—C8	17.8 (6)	C6—C7—C8—Br2	152.3 (4)
C2—C1—C9—O1	80.1 (7)	C6—C7—C8—C9	26.7 (6)
C9—C1—C6—C5	176.8 (5)	Br2—C8—C9—C1	−152.1 (4)
C9—C1—C6—C7	−1.3 (7)	C7—C8—C9—O1	86.0 (6)
C2—C1—C9—C8	−162.7 (6)	C7—C8—C9—C1	−27.5 (6)
C1—C2—C3—C4	−0.8 (10)	Br2—C8—C9—O1	−38.6 (6)

### Hydrogen-bond geometry (Å, º)

**Table:**

*D*—H···*A*	*D*—H	H···*A*	*D*···*A*	*D*—H···*A*
C9—H9···O2	0.98	2.36	2.704 (8)	100
